# Crystal Violet Lactone Salicylaldehyde Hydrazone Zn(II) Complex: a Reversible Photochromic Material both in Solution and in Solid Matrix

**DOI:** 10.1038/srep14467

**Published:** 2015-09-28

**Authors:** Kai Li, Yuanyuan Li, Jing Tao, Lu Liu, Lili Wang, Hongwei Hou, Aijun Tong

**Affiliations:** 1 College of Chemistry and Molecular Engineering, Zhengzhou University, Henan 450001, P. R. China; 2 School of Chemistry and Chemical Engineering, Henan University of Technology, Henan 450001, P. R. China; 3 Department of Chemistry, Beijing Key Lab Microanalytical Methods and Instrumentation, Key Laboratory of Bioorganic Phosphorus Chemistry and Chemical Biology (Ministry of Education), Tsinghua University, Beijing 100084, P. R. China

## Abstract

Crystal violet lactone (CVL) is a classic halochromic dye which has been widely used as chromogenic reagent in thermochromic and piezochromic systems. In this work, a very first example of CVL-based reversible photochromic compound was developed, which showed distinct color change upon UV-visible light irradiation both in solution and in solid matrix. Moreover, metal complex of CVL salicylaldehyde hydrozone was facilely synthesized, exhibiting reversible photochromic properties with good fatigue resistance. It was served as promising solid material for photo-patterning.

Photochromic materials have received considerable attention due to their diverse applications such as optical data storage^1^,^2^,^3^,^4^, photoelectric conversion^5^,^6^,^7^, multifunction photocontrollable switches^8^,^9^,^10^,^11^, photoresponsive smart surfaces^12^,^13^,^14^, molecular machines^15^,^16^, photoresponsive supramolecular self-assembly^17^,^18^, and photocontrollable biological processes^19^,^20^,^21^. Even though a few organic photochromic systems have been well demonstrated including azobenzene, spiropyran, and diarylethenes^22^,^23^, the development of new photochromic system with excellent properties is still in great demand^24^,^25^,^26^,^27^,^28^,^29^,^30^,^31^. Crystal violet lactone (CVL) is a classic triarylmethane (TMP) organic dye with many appealing attributes, such as long-wavelength absorption, high absorption coefficient, high chromaticity, excellent oil-solubility, and low cost^32^. It is widely used as chromogenic reagent in the field of piezochromic and thermochromic materials (e.g. carbonless copy papers and thermal papers) due to its intrinsic halochromic property^32^,^33^. CVL was also reported to be an electrochromic material when it is combined with iron ion: Fe(III) is able to open the lactone of CVL in methanol but Fe(II) can’t^34^. Normally, excessive exposure to light will lead to an irreversible photodegradation for CVL^35^. So far there is however no report of using them as reversible direct photochromic materials. Achievement of photo-sensitive CVL derivatives will greatly expand its potential application areas.

Previous reports have shown that spirolactone-containing dyes exhibited the potential in application of photochromic materials: well-controlled chemical modification of their molecular structures could result in a significant enhancement of photochromic properties, especially in the case of spirolactam formation^1^,^36^,^37^,^38^,^39^,^40^,^41^. In this work, specially designed crystal violet lactone salicylaldehyde hydrazone (**3**) was demonstrated. On one hand, the Schiff-base moiety in salicylaldehyde spirolactam hydrazone is a classic photochromic structure: upon light irradiation, there is a tautomerism from its enol-form to keto-form^42^. On the other hand, salicylaldehyde spirolactam hydrazone is a good chelation group for metal ions. The addition of certain metal ions will lead to a “spirolactam ring-open” reaction along with fluorescence or absorption spectra changes. Several small molecule probes for metal ions have been designed based on this structure^43^. Thus, by introducing salicylaldehyde spirolactam hydrazone to CVL structure (Fig. S1), the CVL derivative (**3**) may have a potential of photo-induced “spirolactam ring-open” properties. In this work, compound **3** was facilely synthesized by two-step synthesis^44^ with all inexpensive reagents. Complex **3-Zn** was obtained by addition of Zn(II) to **3** in dichloromethane (DCM). Excess Zn(II) was used to ensure the efficient formation of the complex.

## Results and Discussion

**Figure 1:**
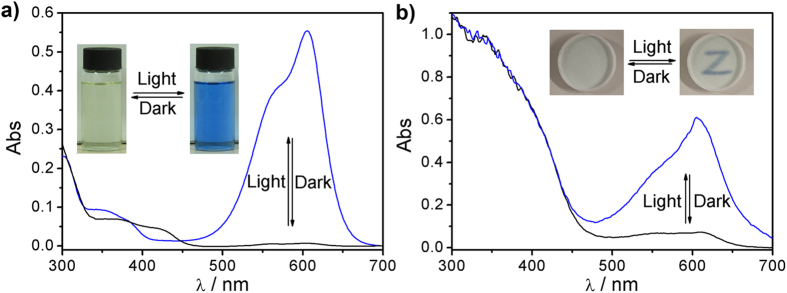
Visible color changes (inset) and the corresponding absorption spectra of **3-Zn** in DCM (**a**) and on silica gel (**b**) before and after light irradiation. Conditions: λ = 405 nm. For (**a**), [**3**] = 10 μmol/L, [Zn(II)] = 100 μmol/L. For (**b**), [**3**] = 10 μmol/g, [Zn(II)] = 100 μmol/g.

Complex **3-Zn** was found to display reversible photochromism in DCM solution. As shown in Fig. 1a and Supplementary Information Video 1, the colorless solution turned blue upon 405 nm light irradiation (the power output of laser point is 20 mW). Moreover, all the conventional light sources even sunlight could induce that photochromic reaction. When light source was removed, the solution gradually restored to its original state. Before light irradiation, almost no absorption band above 450 nm was observed for **3-Zn**. After light irradiation, a strong band centered at 605 nm with a shoulder peak around 567 nm emerged (Fig. 1a). The molar absorption coefficient of **3-Zn** at 605 nm after light irradiation was as high as 5.5 × 10^4^ cm^-1^mol^-1^L, which is similar to that of CVL^45^.

**Figure 2:**
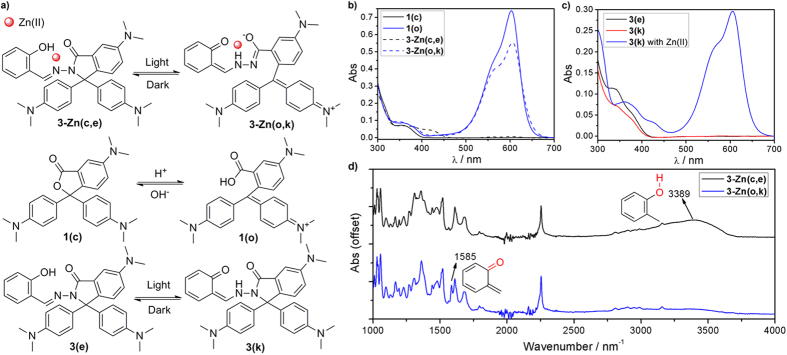
(a) Proposed mechanism for color change of **3-Zn** upon light irradiation (top). The known tautomerism of **1** upon the addition of acid (middle) and **3** upon light irradiation (bottom). (**b**) Absorption spectra of **1** before (**1(c)**) and after (**1(o)**) the addition of 5 equiv HCl, and absorption spectra of **3-Zn** before (**3-Zn(c,e)**) and after (**3-Zn(o,k)**) light irradiation. (**c**) Absorption spectra of **3** before (**3(e)**) and after (**3(k)**) light irradiation, and absorption spectra of **3** after light irradiation, then 10 equiv Zn(II) was added (**3(k)** with Zn(II)). (**d**) Infrared spectra of **3-Zn** before (**3-Zn(c,e)**) and after (**3-Zn(o,k)**) light irradiation. Conditions: λ = 405 nm. For (**b**,**c**), [**1**] = 10 μmol/L, [**3**] = 10 μmol/L, [Zn(II)] = 100 μmol/L. For (**d**) DCM solution with 30% THF was used, [**3**] = 0.1 mol/L, [Zn(II)] = 1 mol/L.

As shown in Fig. 2a, a possible mechanism was proposed to understand the color change of **3-Zn** upon light irradiation. To confirm this assumption, CVL (**1**) was chosen as a control compound. It is well known that **1** is sensitive to pH: the leuco form (**1(c)**, spirolactone ring close) turns to the colored form (**1(o)**, spirolactone ring open) upon the addition of acid in DCM, along with a new absorption band centered at 603 nm^32^,^33^,^34^. The wavelength and shape of the absorption band for **3-Zn** after light irradiation (**3-Zn(o,k)**) were consistent with those of **1(o)** (Fig. 2b), indicating that **3-Zn** underwent a spirolactam ring-open reaction to yield a ring-open product with blue color. As shown in Fig. S2, the binding ratio and binding constant are studied by UV-Vis spectra titration and Job’s plot method. The experimental results show that the binding ratio of **3-Zn** is 1:1 and the binding constant is 6.9 × 10^6^ L/mol.

According to the reports^42^,^46^, light irradiation promoted the tautomerism of salicylaldehyde hydrazone from its enol-form to keto-form. The absorption spectra of **3** showed an obvious decrease in absorbance at 333 nm after light irradiation, which was the result of the tautomerism from the enol-form (**3(e)**) to the keto-form (**3(k)**) (Fig. 2c, black line and red line). Meanwhile, no absorption band at 605 nm could be observed, indicating that the spirolactam in **3** was photostable without Zn(II). These results demonstrate that Zn(II) is an indispensable factor for the photochromism of **3-Zn**. Interestingly, a characteristic absorption band centered at 605 nm emerged upon the addition of Zn(II) to the pre-exposed **3** (**3(k)**) (Fig. 2c, blue line), which further supported the existence of **3-Zn(o,k)**. In addition, more intuitive evidence was from the fluorescence spectra. **3-Zn** exhibits strong fluorescence which is attributed to the chelation-enhanced fluorescence (CHEF) of salicylaldehyde hydrazone with Zn(II)^47^. After light irradiation, **3-Zn** showed a weaker fluorescence than its precursor (Fig. S3). This fluorescence decrease was attributed to the formation of keto-form that can serve as a strong fluorescence quenching unit.

As shown in Table 1, the light source to promote photochromism of **3-Zn** was at least within 325 nm to 450 nm. This range was fitted well with the absorption band of **3-Zn(c,e)** (Fig. 1a). Normally, single benzene ring exhibited no absorption band above 300 nm, so the absorption band of **3-Zn(c,e)** was mainly attributed to the absorbance of salicylaldehyde hydrazone Zn(II) complex moiety. These results further suggested that the salicylaldehyde hydrazone Zn(II) complex moiety was the light response group.

Furthermore, a more direct evidence for the tautomerism of **3-Zn** from the enol-form to the keto-form was observed from the infrared spectra. As shown in Fig. 2d, **3-Zn(c,e)** showed a broad absorption peak at 3389 cm^-1^ which is assigned as a stretching mode of the phenolic hydroxyl group. After light irradiation, the broad peak decreased and a new peak at 1585 cm^−1^ emerged which is assigned as a stretching mode of the new carbonyl group in **3-Zn(o,k)**.

According to the above experimental results, the mechanism of photochromism of **3-Zn** should be as follows: light irradiation promoted the tautomerism of salicylaldehyde hydrazone moiety in the complex from its enol-form (**3-Zn(c,e)**) to keto-form (**3-Zn(o,k)**), and this change enhanced the chelation of Zn(II) and induced spirolactam ring-open reaction in **3-Zn** to yield a blue-colored product (Fig. 2a).

**Figure 3:**
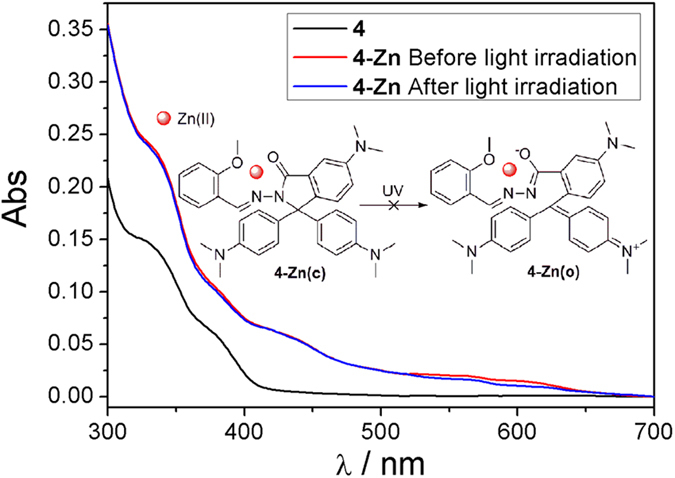
Absorption spectra of **4** and **4-Zn** before and after light irradiation. Conditions: λ = 405 nm. [**4**] = 10 μmol/L, [Zn(II)] = 100 μmol/L.

To further testify the proposed mechanism, a control compound **4** containing a methoxy group instead of the phenolic hydroxyl group (Fig. 3 inset) was prepared and examined. As shown in Fig. 3, the changes of absorption spectra indicated that **4** was able to chelate Zn(II) to form **4-Zn**. However, **4-Zn** complex had no photochromic property upon light irradiation. These results prove that the tautomerism of phenolic hydroxyl group is an indispensable factor for the photochromism.

As shown in Fig. S4, different metal ions were used to replace Zn(II) in complex **3-Zn**. The photochromic properties couldn’t be observed in most of the common metal ions except for Cd(II), which is in the same group of the periodic table with Zn(II) and usually induces a similar coordination property. However, since Cd(II) is highly toxic for organism, it is not suitable for the development of environment-friendly materials.

Moreover, density functional theory (DFT) calculations were performed on **3-Zn(c,e)** and **3-Zn(o,k)** with Gaussian 09 program to understand their electronic structure. B3LYP hybrid density function and the 6-31G (d,p) basis set were used to optimize the geometries. As shown in Fig. S5, LUMO energy level are distributed on the salicylaldehyde hydrazone Zn(II) complex moiety and HOMO energy level are distributed on the dimethylaminophenyl group for both **3-Zn(c,e)** and **3-Zn(o,k)**. These results suggest that the salicylaldehyde hydrazone Zn(II) complex moiety is an active group in the photochromic reaction, supporting the proposed mechanism.

**Figure 4:**
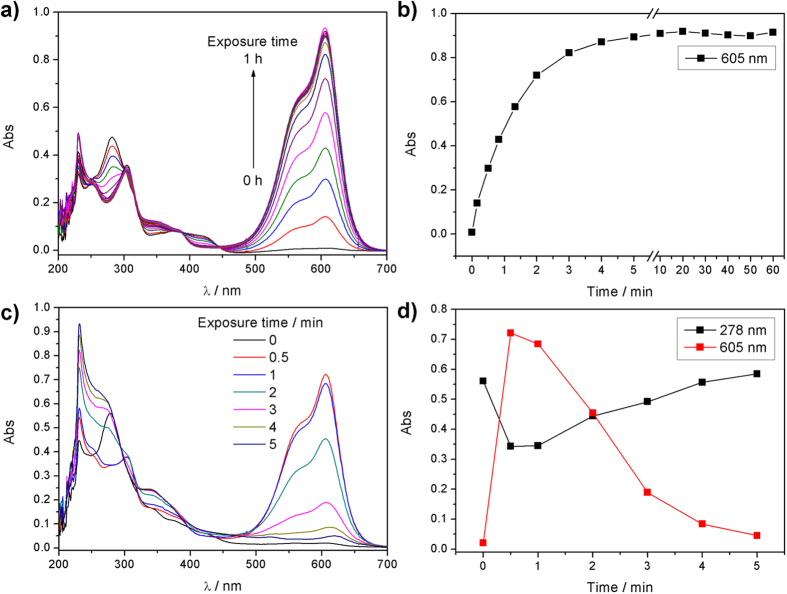
Absorption spectra of **3-Zn** upon the irradiation different light source. Conditions: [**3**] = 10 μmol/L, [Zn(II)] = 100 μmol/L. For (**a**,**b**), a 6 W hand-held UV lamp at 365 nm was used. For (**c,d**), a 500 W mercury lamp was used.

The response time and photostability depend on the intensity of the light source. As shown in Fig. 4a,b, the response time of **3-Zn** is about 5 min upon the irradiation of a 6 W hand-held UV lamp at 365 nm. After being uninterrupted irradiated for 1 h, the absorption spectra of **3-Zn** barely change, indicating a good stability of **3-Zn** under common light source irradiation. When **3-Zn** is irradiated by high-intensity light of a 500 W mercury lamp, the response time is less than 30 s (Fig. 4c,d). Excessive exposure to high-intensity light will lead to an irreversible photodegradation: the absorption band around 605 nm gradually disappear when the exposure time is longer than 30 s under a 500 W mercury lamp, while a strong band from 230 nm to 300 nm emerges. After being irradiated for 5 min, the absorption band around 605 nm almost disappears, indicating that **3-Zn** has been degraded completely by light. The new absorption band from 230 nm to 300 nm was attributed to the small molecular degraded products.

The reversibility and fatigue resistance of **3-Zn** are both important factors in its performance as potential photochromic materials. Since there is no absorption band around 605 nm for **3-Zn(c,e)**, the absorbance at 605 nm is proportional to the concentration of **3-Zn(o,k)**. As shown in Fig. S6, 10 μmol/L of light exposed **3-Zn** turns to its leuco form in about 1 h at 25 °C. Moreover, the decay curve could be fitted well with first-order reaction kinetics (ln A = −*k*t), indicating that the transition from **3-Zn(o,k)** to **3-Zn(c,e)** is a first-order reaction (Fig. S6 inset). Therefore, the recovery rate is proportional to the concentration of **3-Zn(o,k)**. The thermal bleaching speed constant and half-life of **3-Zn(o,k)** at room temperature are calculated to be 1.36 × 10^−3^ s^−1^ and 510 s, respectively, which is analogous to some photochromic naphthopyran compounds^48^. Besides the reversibility, the fatigue resistance of **3-Zn** was also investigated. As shown in Fig. S7a, **3-Zn** was toggled repeatedly between the leuco state and colored state for 10 times while the absorbance at 605 nm stayed constant without degradation.

**Figure 5:**
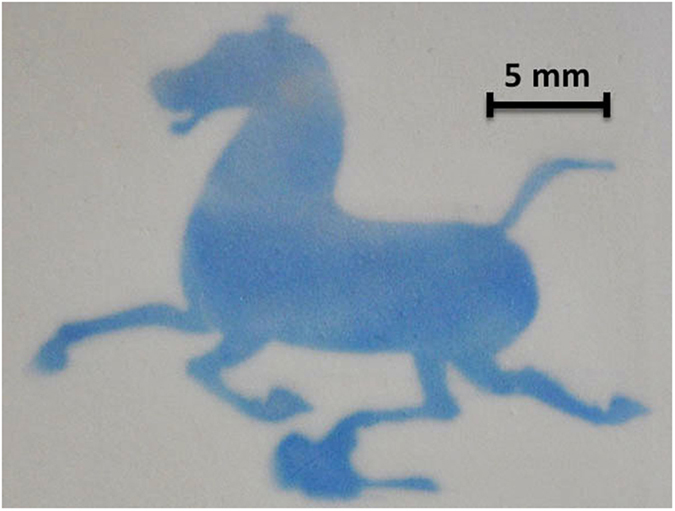
Generating patterns on **3-Zn** on silica gel plate upon light irradiation. Conditions: λ = 405 nm. [**3**] = 10 μmol/g, [Zn(II)] = 100 μmol/g.

**3-Zn** also displayed similar color and spectral changes in solid matrix such as silica gel (Fig. 1b). The fatigue resistance of **3-Zn** on silica gel was as good as it in DCM (Fig. S7b). In silica gel, the light exposed product does not return exactly to the starting point, which might be due to the following reason: silica gel is acidic, in which the proton will lead to a side reaction for **3-Zn** (Fig. S8)^49^,^50^,^51^. Fortunately, this flaw doesn’t impact the application of **3-Zn** for solid photochromic material. As shown in Fig. 5 and Supplementary Information Video 2, pattern is successfully visualized with the highest resolution up to 0.5 mm, which indicate that **3-Zn** could be served as promising solid material for photo-patterning.

## Conclusions

In conclusion, a new TMP organic dye-based photochromic system has been developed for the first time. Crystal violet lactone salicylaldehyde hydrazone Zn(II) complex showed a distinct color change upon light irradiation in the UV-visible range. The mechanism studies showed that light irradiation promoted tautomerism of the phenolic hydroxyl group in salicylaldehyde hydrazone moiety from the enol-form to the keto-form, and subsequently enhanced the chelation of Zn(II), inducing a spirolactam ring-open reaction to yield a blue-colored product. The photochromic reaction was reversible with good fatigue resistance. More importantly, the system exhibited photochromic property not only in solution but also in solid matrix, which makes it promising for photo-patterning applications. This work expands the spirolactone-containing dye photochromic family and will greatly further the advancement of CVL applications. Efforts on the development of more TMP-based photochromic system are in progress in our laboratories.

## Methods

### General

In these experiments, all the materials of analytical grade were used without further purification. Crystal violet lactone, hydrazine hydrate, salicylaldehyde, 2-methoxybenzaldehyde and zinc nitrate hexahydrate were purchased from J&K Chemical Co., Beijing, China. All the other materials were purchased from Sinopharm Chemical Reagent Beijing Co., Beijing, China. Absorption spectra were measured by JASCO V-550 UV-Vis spectrophotometer, 1 cm and 0.2 quartz cells are used, respectively. UV-Vis diffuse reflectance spectra were measured by Hitachi U-3010 UV-Vis spectrophotometer, BaSO_4_ was used as reference. Fluorescence spectra were determined on Hitachi F-4500 fluorescence spectrometer. Infrared spectra were performed on a Perkin-Elmer Frontier FT-IR/NIR spectrometer with a universal attenuated total reflection sampling accessory. NMR spectra were recorded by JOEL JNM-ECA400 spectrometer operated at 400 MHz and Bruker AVANCE III spectrometer operated at 600 MHz. ESI-MS spectra were obtained on SHIMADZU LCMS-IT-TOF LC-MS spectrometer without using the LC part. Laser of 325 nm was produced by Kimmon series He-Cd laser, laser of 375 nm was produced by CNI MDL-III-375 laser, laser of 405 nm was produced by LD-T405F00 laser pointer (power output 20 mW), laser of 450 nm laser was produced by CNI GLP-450 laser, laser of 532 nm was produced by FONLIN G-301 laser pointer. The photos and videos were carried with Canon EOS 600D camera.

### Synthesis and characterization

The synthetic route to the target compounds is shown in Fig. S1. The compounds structures were verified by NMR and ESI-MS. The original data of NMR spectra and ESI-MS spectra were given in Supplementary Information.

#### Crystal violet lactone hydrazide (2)

To a 100 mL flask, crystal violet lactone (830 mg, 2 mmol) was dissolved in a mixed solvent of DCM (20 mL) and absolute ethanol (20 mL). After the addition of hydrazine hydrate (80%, 3 mL, excess), the stirred mixture was heated to 90 °C for 3 h. Then the solvent was concentrated to 10 mL under reduced pressure. After that, the solution was cooled to 4 °C to yield a white precipitate. The resulting precipitate was filtrated and washed with 30 mL absolute ethanol for three times. After dried under reduced pressure, 620 mg **2** (yield 73%) was obtained as white solid. ESI-MS spectrometry: *m/z* for [*M*+H]^+^, calculated: 430.2607, found: 430.2601. ^1^H NMR (DCCl_3_) δ (ppm): 2.91 (s, 12H), 2.99 (s, 6H), 4.82 (s, 1H), 6.62 (d, 4H, *J* = 8.7 Hz), 6.77 (m, 2H), 7.02 (d, 4H, *J* = 8.7 Hz), 7.08 (s, 1H), 7.41 (d, 1H, *J* = 2.3 Hz). ^13^C NMR (DCCl_3_) δ (ppm): 40.57, 40.68, 67.65, 110.84, 112.11, 115.47, 128.04, 128.83, 129.65, 130.78, 134.54, 149.52, 149.70, 166.11.

#### Salicylaldehyde crystal violet hydrazone (3)

To a 100 mL flask, **2** (429 mg, 2 mmol) and salicylaldehyde (305 mg, 2.5 mmol) were dissolved in a mixed solvent of DCM (20 mL) and absolute ethanol (20 mL). The stirred mixture was heated to 90 °C for 3 h. Then the solvent was concentrated to 10 mL under reduced pressure. After that, the solution was cooled to 4 °C to yield a yellow precipitate. The precipitate was purified by column chromatography on silica gel (petroleum ether/EtOAc, 3:1) to give 219 mg **3** (yield 41%) as a yellow solid. ESI-MS spectrometry: *m/z* for [*M*+H]^+^, calculated: 534.2869, found: 534.2864. ^1^H NMR (DCCl_3_) δ (ppm): 2.88 (s, 12H), 2.97 (s, 6H), 6.62 (d, 4H, *J* = 8.7 Hz), 6.85 (m, 2H), 7.13 (d, 4H, *J* = 8.7 Hz), 7.17 (m, 3H), 7.32 (d, 1H, *J* = 2.3 Hz), 9.81 (s, 1H), 10.83 (s, 1H). ^13^C NMR (DCCl_3_) δ (ppm): 40.47, 40.82, 75.72, 105.81, 112.12, 116.91, 117.90, 118.86, 119.24, 124.34, 128.38, 129.25, 130.36, 131.49, 131.81, 137.65, 149.96, 150.69, 155.91, 158.75, 166.08.

#### 2-Methoxybenzaldehyde crystal violet hydrazone (4).

To a 100 mL flask, **2** (429 mg, 2 mmol) and 2-methoxybenzaldehyde (340 mg, 2.5 mmol) were dissolved in a mixed solvent of DCM (20 mL) and absolute ethanol (20 mL). The stirred mixture was heated to 90 °C for 3 h. Then the solvent was concentrated to 10 mL under reduced pressure. After that, the solution was cooled to 4 °C to yield a light green precipitate. The resulting precipitate was filtrated and washed with 30 mL absolute ethanol for three times. After dried under reduced pressure, 339 mg **4** (yield 62%) was obtained as light green solid. ESI-MS spectrometry: *m/z* for [*M*+H]^+^, calculated: 548.3026, found: 548.3016. ^1^H NMR (DCCl_3_) δ (ppm): 2.89 (s, 12H), 2.98 (s, 6H), 3.81 (s, 3H), 6.61 (d, 4H, *J* = 8.7 Hz), 6.85 (m, 3H), 7.18 (m, 5H), 7.26 (m, 1H), 7.84 (d, 1H, *J* = 6.0 Hz), 10.04 (s, 1H). ^13^C NMR (DCCl_3_) δ (ppm): 40.55, 40.86, 55.66, 75.62, 106.03, 111.05, 111.78, 117.35, 120.58, 124.48, 124.78, 126.21, 129.12, 129.65, 130.94, 131.15, 137.39, 147.48, 149.66, 150.58, 158.43, 165.87.

### Preparation of photochromic system

1 mol/L Zn(NO_3_)_2_ stock solution was prepared in THF. 0.01 mol/L **3** or **4** stock solution was prepared in DCM. The photochromic system in solution was prepared by the addition of 10 equivalent Zn(II) to the solution of **3** in corresponding concentration. The photochromic system on silica gel was prepared as following: 10 g silica gel (200–300 mesh), 1 mL 1 mol/L Zn(NO_3_)_2_ stock solution and 10 mL 0.01 mol/L **3** stock solution were added to 30 mL DCM solution. After well mixed, DCM was evaporation under reduced pressure at room temperature. The products were obtained as light green powders.

## Additional Information

**How to cite this article**: Li, K. *et al.* Crystal Violet Lactone Salicylaldehyde Hydrazone Zn(II) Complex: a Reversible Photochromic Material both in Solution and in Solid Matrix. *Sci. Rep.* **5**, 14467; doi: 10.1038/srep14467 (2015).

## Supplementary Material

Supplementary Information

Supplementary Video 1

Supplementary Video 2

## Figures and Tables

**Table 1:** The influence of different wavelength light to the color change of **3-Zn.**

**Wavelength/nm**	**325**	**375**	**405**	**450**	**532**
Color change	yes	yes	yes	yes	no
