# Supplementary material for: Crystal Violet Lactone Salicylaldehyde Hydrazone Zn(II) Complex: a Reversible Photochromic Material both in Solution and in Solid Matrix
Source: Sci Rep. 2015 Sep 28;5:14467. doi: 10.1038/srep14467 (PMC4585988; doi:10.1038/srep14467)
Supplement: Supplementary Information [file srep14467-s1.pdf]

Crystal Violet Lactone Salicylaldehyde Hydrazone Zn(II) Complex: a Reversible Photochromic Material both in Solution and in Solid Matrix

Kai Li,[1] Yuanyuan Li,[2] Jing Tao,[3] Lu Liu,[1] Lili Wang,[1] Hongwei Hou[1] and Aijun Tong[3]

[1] College of Chemistry and Molecular Engineering, Zhengzhou University, Henan 450001, P. R. China

[2] School of Chemistry and Chemical Engineering, Henan University of Technology, Henan 450001, P. R. China

[3] Department of Chemistry, Beijing Key Lab Microanalytical Methods and Instrumentation, Key Laboratory of Bioorganic Phosphorus Chemistry and Chemical Biology (Ministry of Education), Tsinghua University, Beijing 100084, P. R. China

Correspondence and requests for materials should be addressed to K. L. (likai@zzu.edu.cn) or A. T. (tongaj@mail.tsinghua.edu.cn)

Contents

1. Selected spectra and data referred in the paper.. S2

2. Caption of videos.. S4

3. NMR spectra and ESI-MS spectra... S5

1. Selected spectra and data referred in the paper

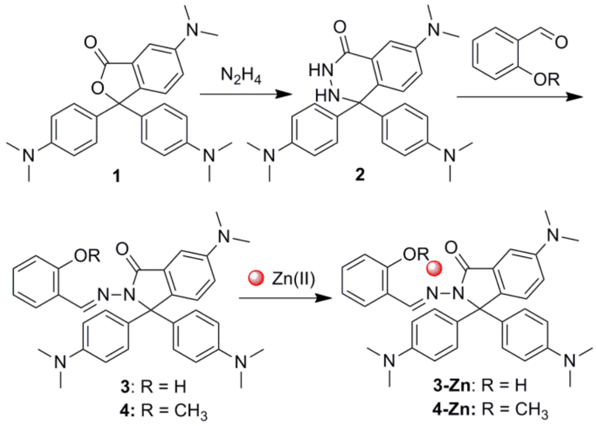

Figure S1. Synthesis of **3-Zn** and **4-Zn.**

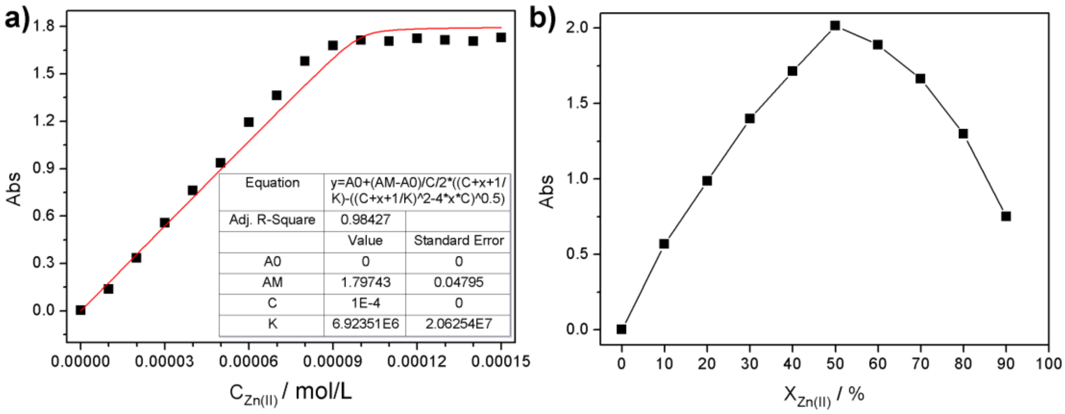

Figure S2. The results of UV-Vis spectra titration and Job's plot method. Conditions: the mixture of **3** and Zn(II) was irradiated by an 6 W hand-held UV lamp at 365 nm for 5 min before test. Absorption λ = 605 nm. For a), [**3**] = 100 μmol/L, [Zn(II)] = 0-150 μmol/L, 2 mm quartz cells. For b) [**3**] + [Zn(II)] = 100 μmol/L, 1 cm quartz cells.

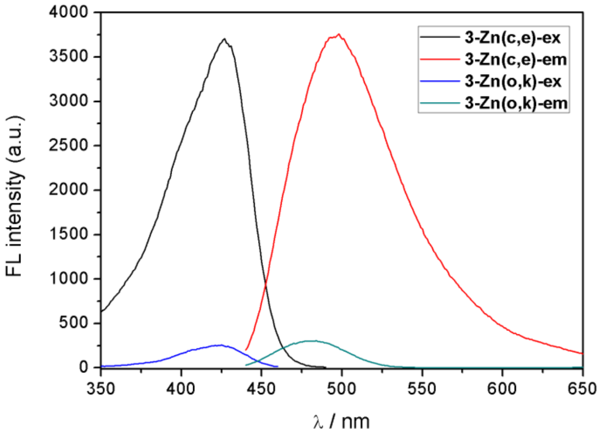

Figure S3. Fluorescence spectra of **3-Zn** before and after UV irradiation. Conditions: [**3**] = 10 μmol/L, [Zn(II)] = 100 μmol/L.

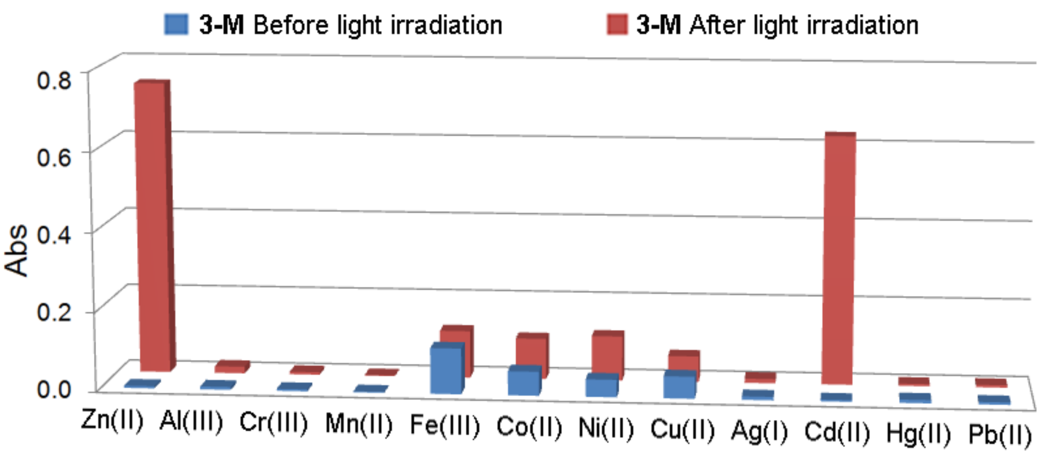

Figure S4. Compound **3** in the presence of different metal ions before and after UV irradiation. Conditions: absorption λ = 605 nm. [**3**] = 10 μmol/L, [M] = 100 μmol/L.

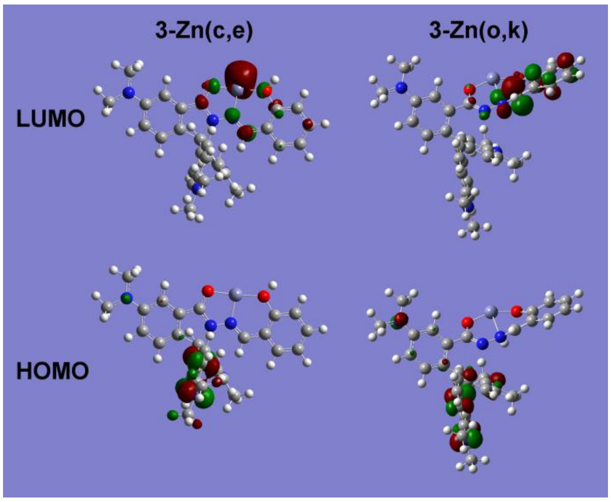

Figure S5. Frontier canonical Kohn-Sham valence MO envelopes of **3-Zn(c,e)** and **3-Zn(o,k)**, with dominant AO contributions. Value of contour envelopes is 0.05 au.

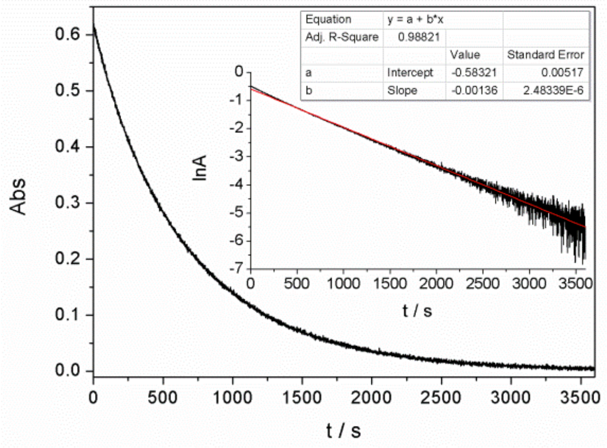

Figure S6. The thermal fading kinetics of **3-Zn** in DCM at 25 ^{o}C. Inset: The curve fitted with one-order reaction dynamics (ln A = -*k*t), the thermal bleaching speed constant (*k*) was calculated to be 1.36×10^{-3} s^{-1}. [**3**] = 10 μmol/L, [Zn(II)] = 100 μmol/L, λ = 609 nm.

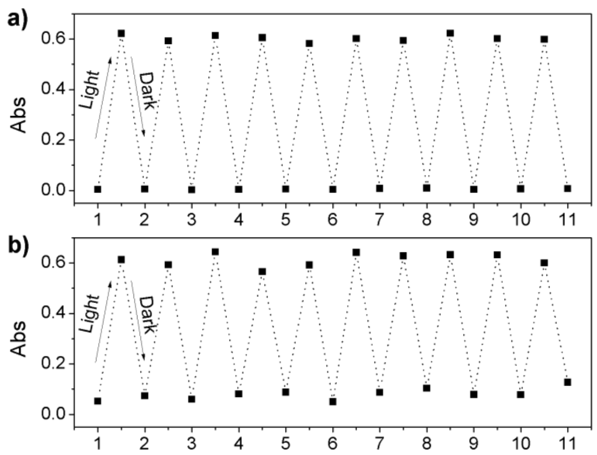

Figure S7. Fatigue resistance of **3-Zn** in DCM (a) and on silica gel (b) upon light irradiation and stand in dark alternatively.

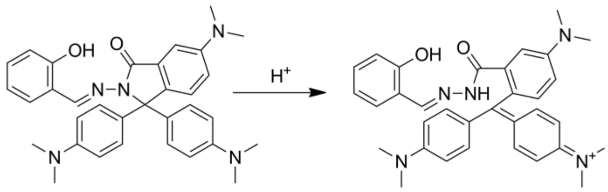

Figure S8. The possible side reaction of **3-Zn** in silica gel.

2. Caption of videos

Video 1: A video of photochromism of **3-Zn** in DCM at room temperature. [**3**] = 10 μmol/L, [Zn(II)] = 100 μmol/L, wavelength of laser is 405 nm.

Video 2: A video of letters recording on **3-Zn** in silica gel at room temperature. [**3**] = 10 μmol/g, [Zn(II)] = 100 μmol/g, wavelength of laser is 405 nm.

3. NMR spectra and ESI-MS spectra

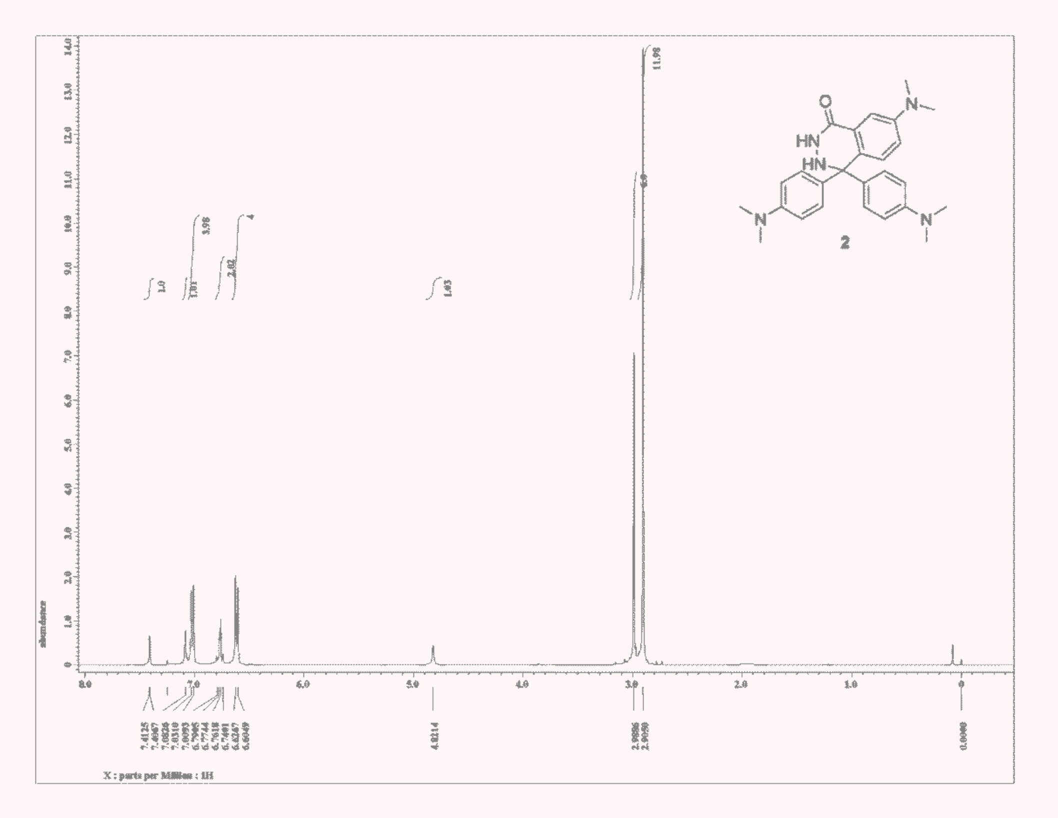

Figure S9. ^{1}H-NMR spectrum of **2** in $DCCl_3$.

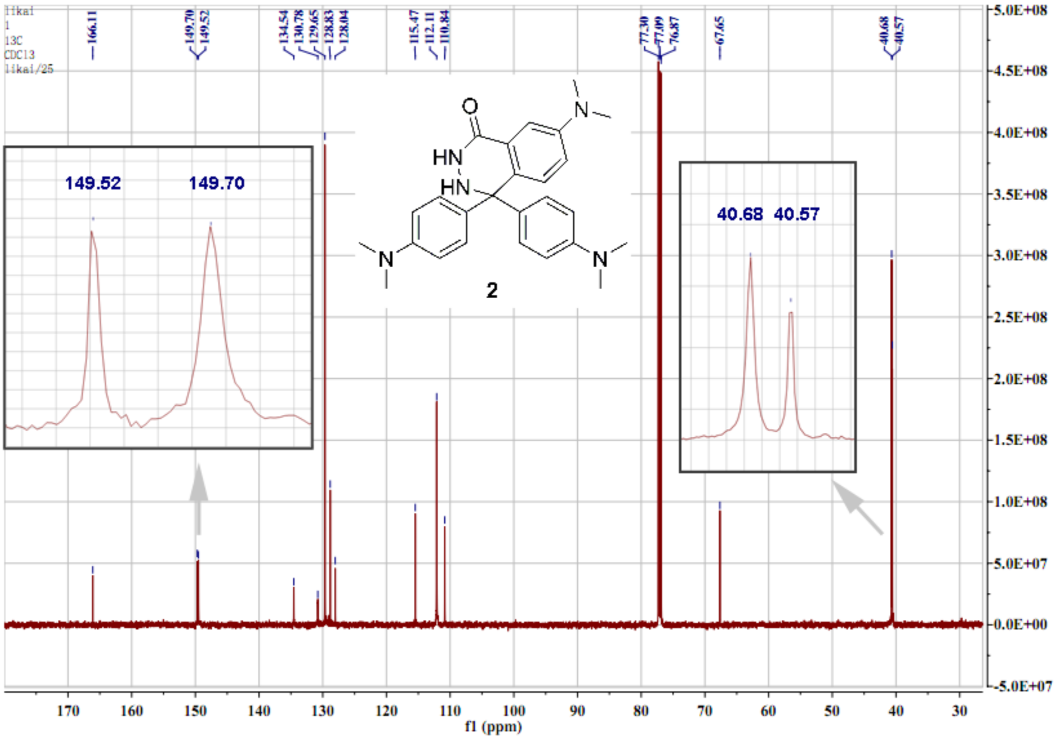

Figure S10. ^{13}C-NMR spectrum of **2** in $DCCl_3$ (Recorded by Bruker AVANCE III spectrometer operated at 600 MHz).

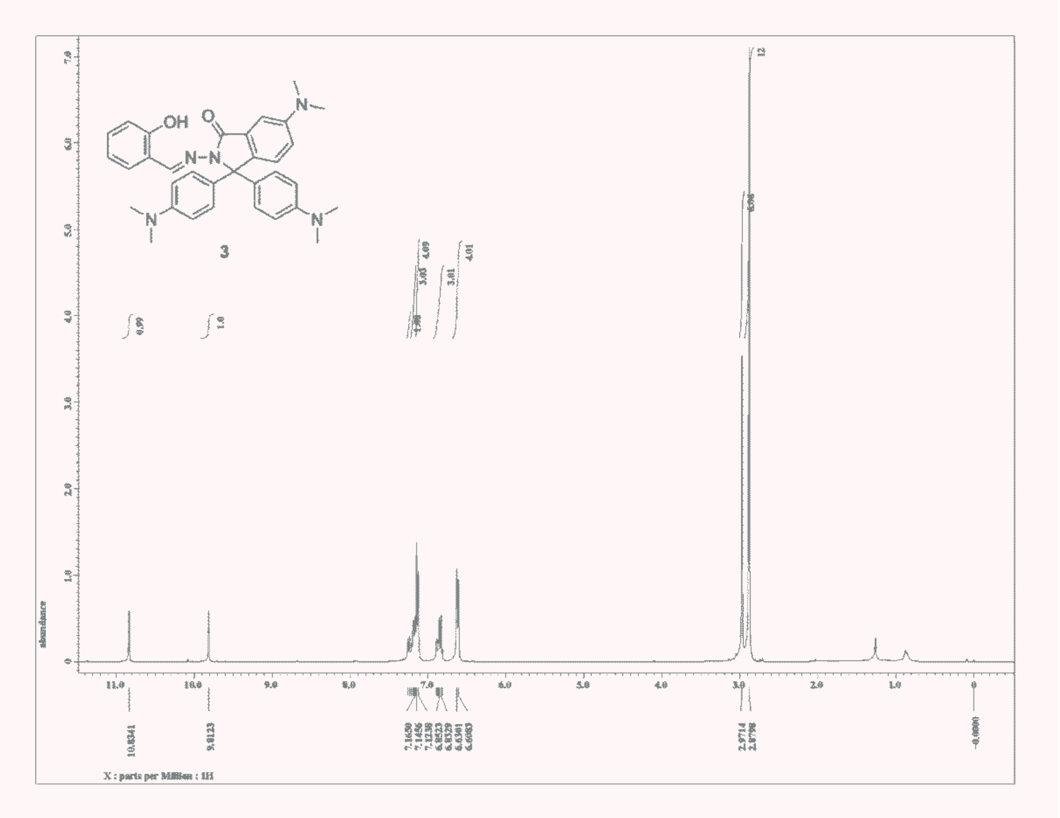

Figure S11. ^{1}H-NMR spectrum of **3** in $DCCl_3$.

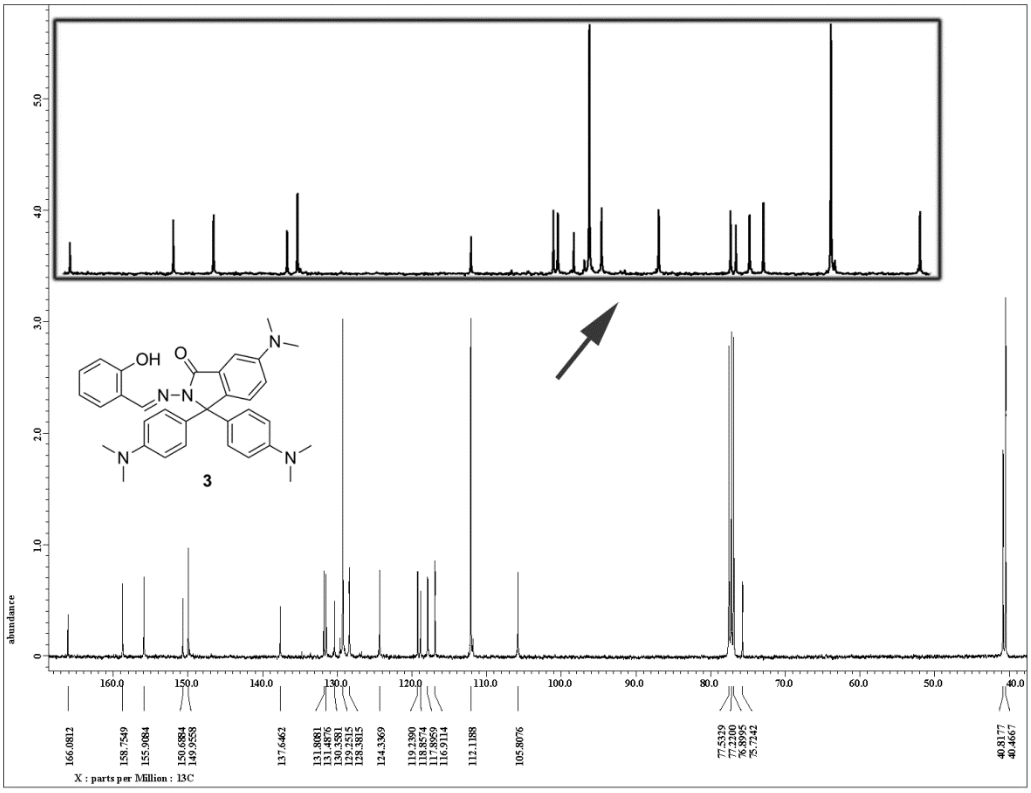

Figure S12. ^{13}C-NMR spectrum of **3** in $DCCl_3$.

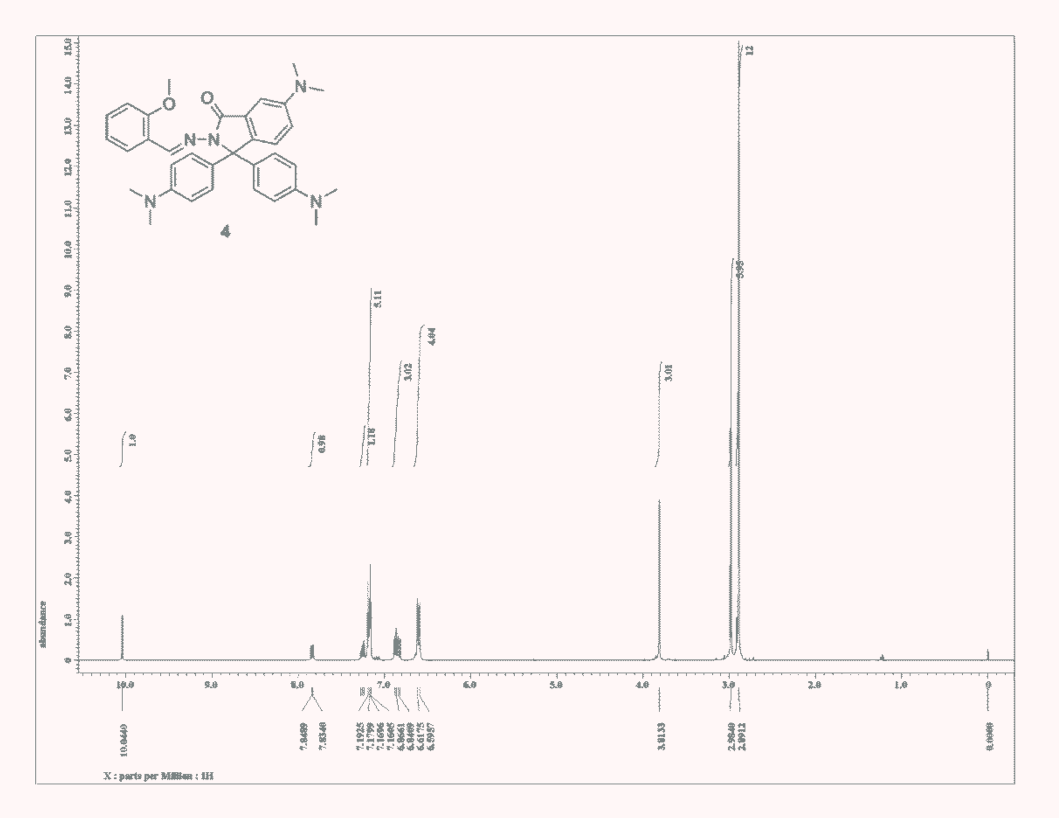

Figure S13. ^{1}H-NMR spectrum of **4** in $DCCl_3$.

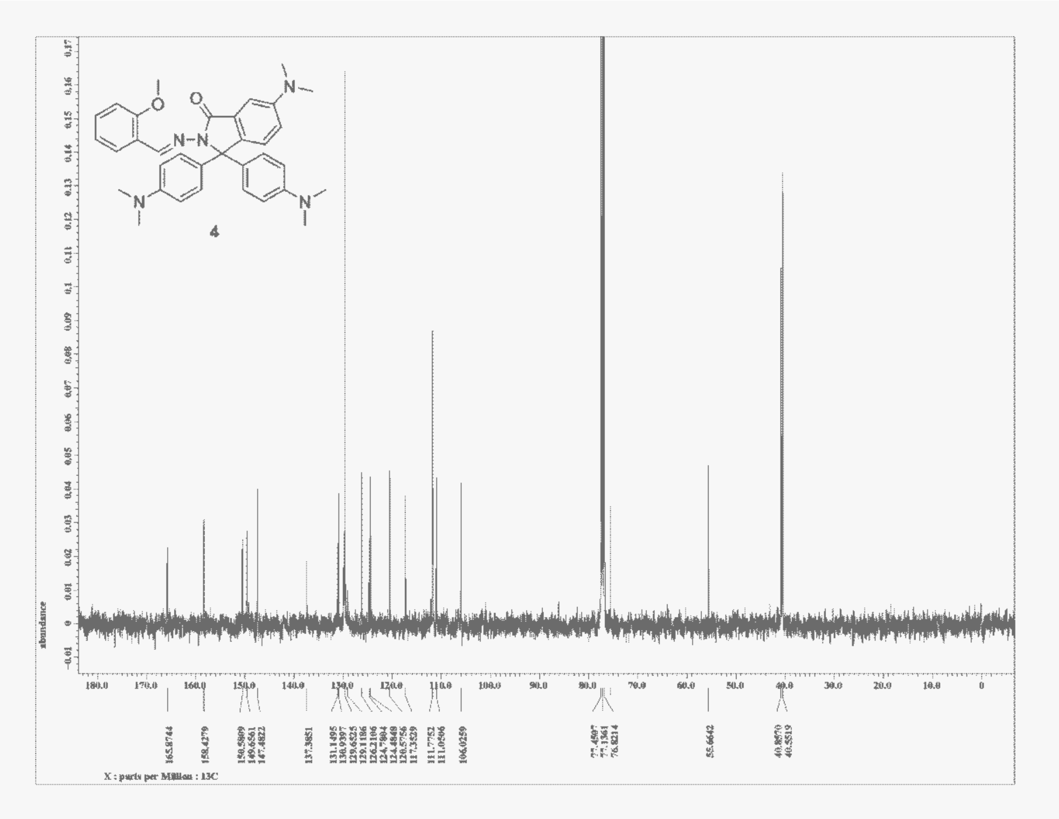

Figure S14. ^{13}C-NMR spectrum of **4** in $DCCl_3$.

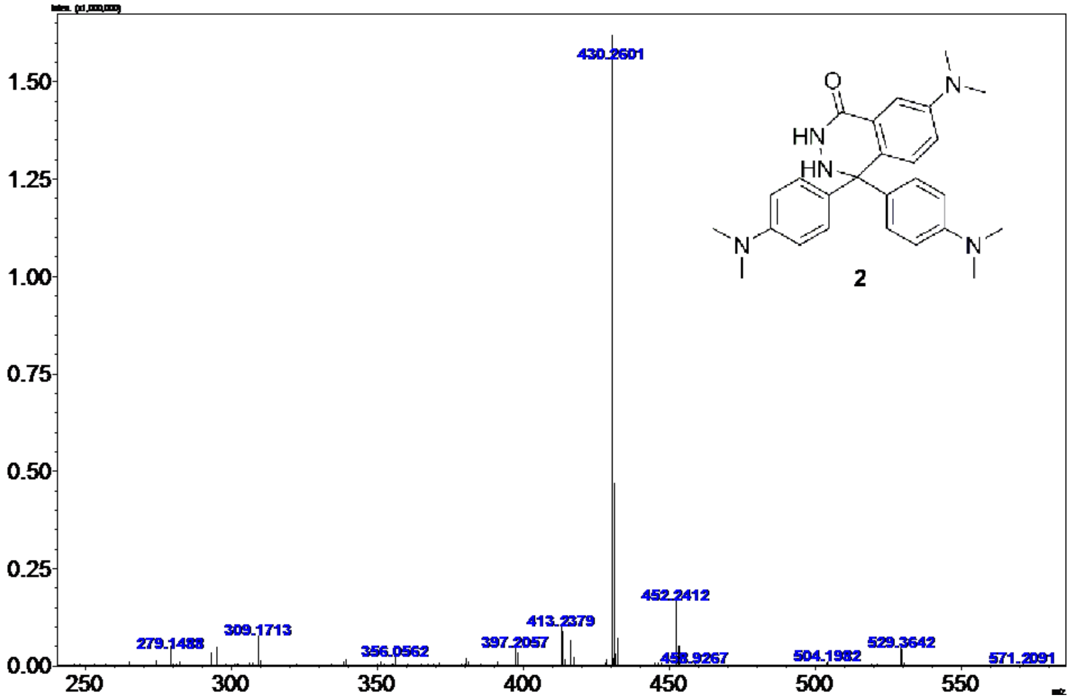

Figure S15. ESI-MS spectrum of **2**.

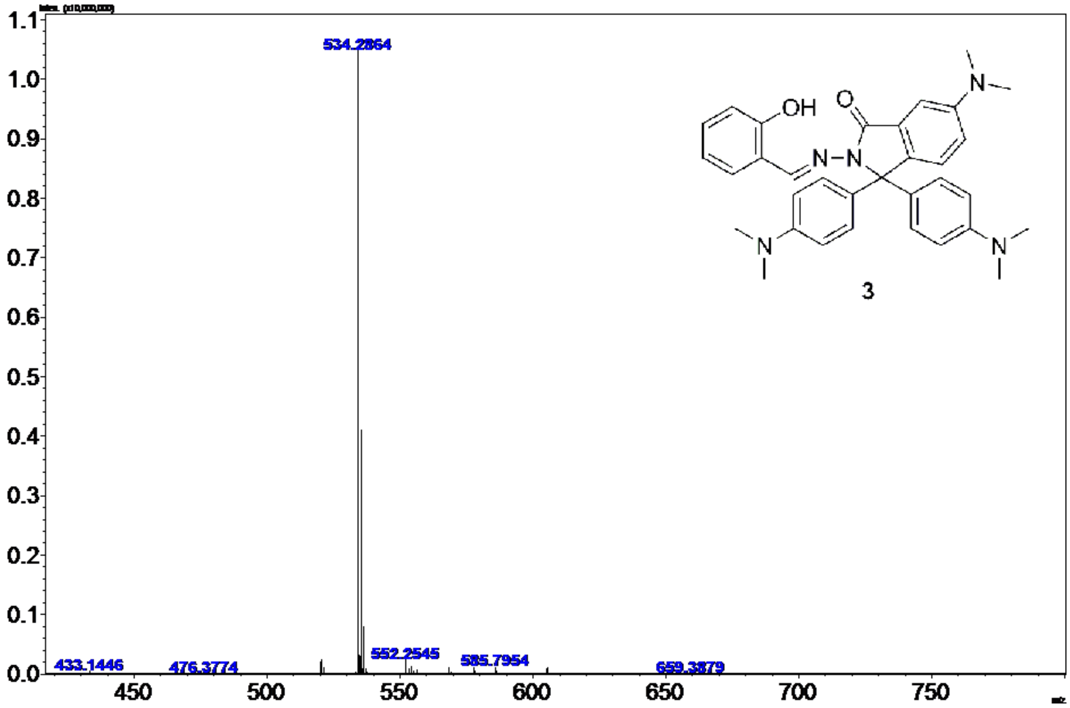

Figure S16. ESI-MS spectrum of **3**.

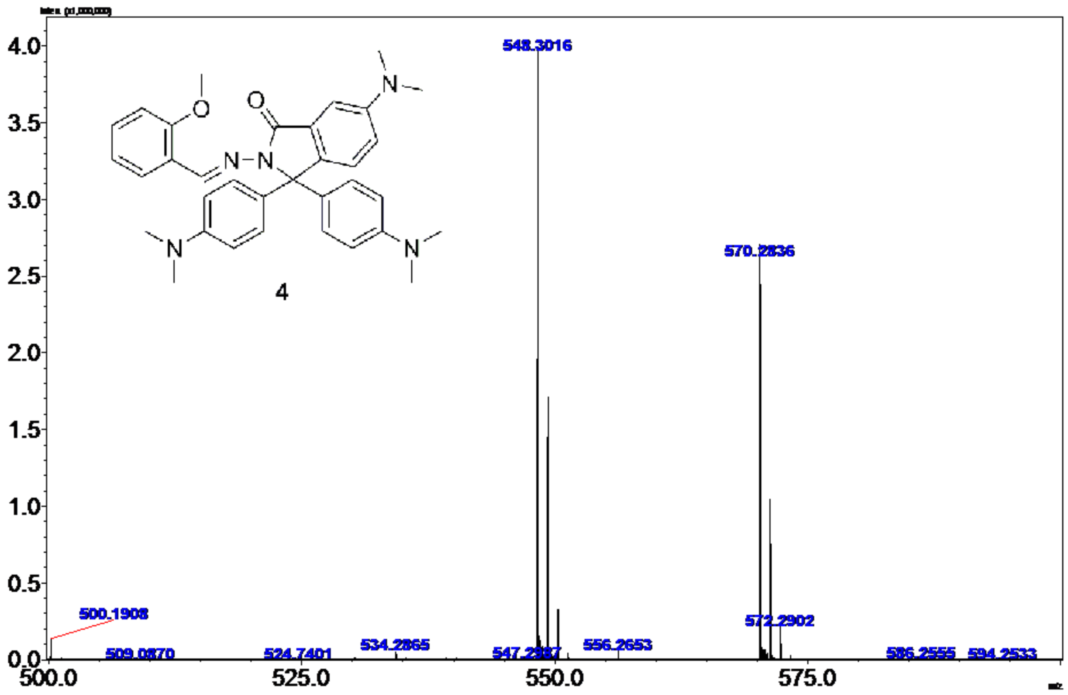

Figure S17. ESI-MS spectrum of **4**.
